# Rare case report: primary small-cell neuroendocrine carcinoma of the gallbladder

**DOI:** 10.3389/fonc.2025.1524974

**Published:** 2025-04-03

**Authors:** Haichen Wang, Qinglin Li, Shaoqi Han, Hu Tian

**Affiliations:** Department of General Surgery, The First Affiliated Hospital of Shandong First Medical University & Shandong Provincial Qianfoshan Hospital, Jinan, China

**Keywords:** gallbladder, small-cell neuroendocrine carcinoma, case report, diagnosis, treatment

## Abstract

Gallbladder cancer (GBC) accounts for 1.7% of all cancer-related deaths. Neuroendocrine carcinoma of the gallbladder (GB-NEC) is a rare subtype of GBC that is more malignant than GBC. Small-cell neuroendocrine carcinoma of the gallbladder (GB-SCNEC) is a rare malignant tumor with a low incidence. To date, no universally accepted or satisfactory treatment exists. This case report details the clinical presentation, diagnostic process, and treatment strategy of a patient with GB-SCNEC. The analysis of this rare case is intended to provide clinicians with diagnostic and therapeutic insights for future research.

## 1 Introduction

**Figure 1:**
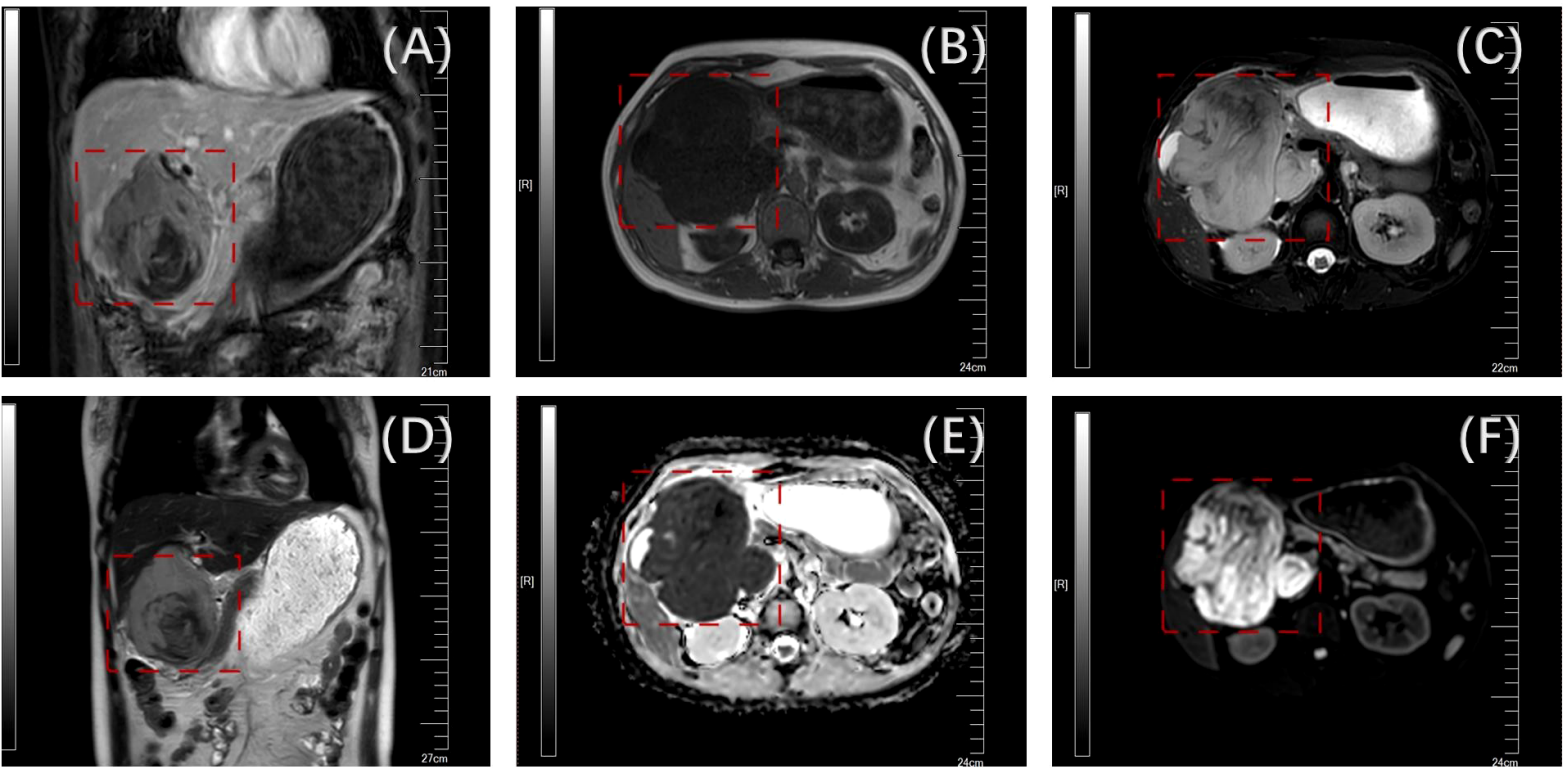
Preoperative enhanced nuclear magnetic examination(the tumor is boxed) [**(A)** mDIXON; **(B)** sF; **(C)** DWI; **(D)** T2W; **(E)** dADC; **(F)** T2].

A 39-year-old woman was admitted to our hospital with intermittent abdominal distention and nausea for 3 weeks. Upon admission, the patient underwent a thorough examination and evaluation. The primary symptoms were abdominal distension, nausea, vomiting (of gastric contents), fatigue, and poor appetite. The patient had no previous history of hypertension, diabetes mellitus, coronary atherosclerotic heart disease, infectious disease, cesarean section, or familial disease. Visual examination revealed no yellow staining of the skin, mucous membranes, or sclera, a flat abdomen, no gastrointestinal pattern or peristaltic wave, and no abdominal wall varicose veins. Palpation revealed a soft abdomen, a palpable tough mass in the right upper abdomen, tenderness, no rebound pain, no clear mass, and a negative Murphy’s sign (-). The liver and spleen were not palpable on examination. Percussion revealed tenderness in the hepatic region, no obvious tenderness in the renal region, and no mobile or turbid sounds. Auscultation revealed weak bowel sounds. An enhanced computed tomography (CT) scan of the abdomen was performed at a local hospital, suggesting hepatic space-occupying lesions that might involve the hepatoportal area and enlarged retroperitoneal lymph nodes. The nature of the lesion could not be determined based on the CT results, and the possibility of a malignant tumor could not be ruled out. Therefore, we performed an MR+MRCP to evaluate the nature of the lesion. The results suggested a huge mass in the gallbladder area, approximately 10.1 × 9.5 cm, marked enhancement on enhancement scan, and delayed enhancement. The lesion encroached on the liver and the sinusoidal portion of the stomach, and the initial duodenal segment and pancreatic head were pushed and shifted. The hepatic hilar and retroperitoneal areas were enlarged with multiple lesions. The preoperative diagnoses included a space-occupying lesion in the gallbladder region, gastric retention, upper gastrointestinal obstruction, possibility of a secondary malignant tumor in the lymph nodes in the hilar region, and possibility of a secondary malignant tumor in the retroperitoneal lymph nodes (**Figure 1**).

**Figure 2:**
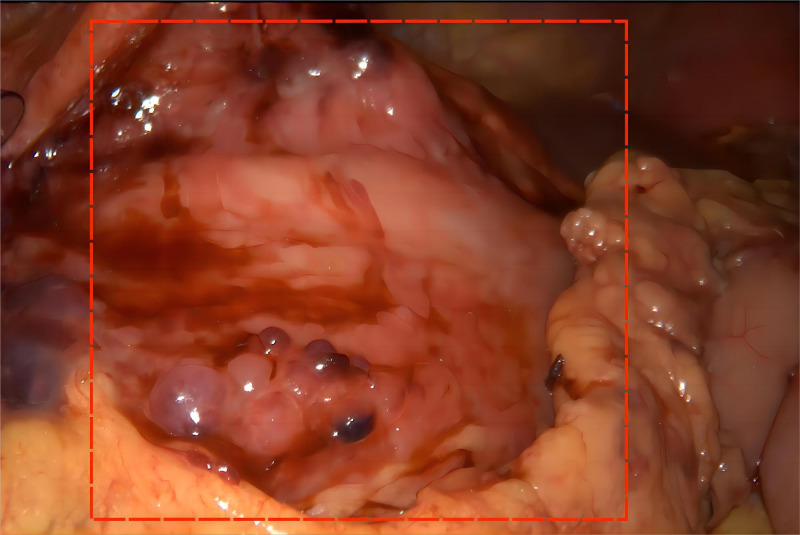
Intraoperative images (intraoperative tumor location is boxed).

After communicating with the patient and his family to relieve gastrointestinal obstruction and clarify the nature of the pathology, laparoscopy, laparoscopic gastrojejunostomy, and puncture biopsy of the liver tumor were performed under general anesthesia 11 days after admission on June 25, 2024. During the operation, the tumor was found to be large, compressing the duodenum and invading the gastric sinus and other neighboring organs; therefore, a tumor biopsy was performed to clarify the pathology (**Figure 2**).

**Figure 3:**
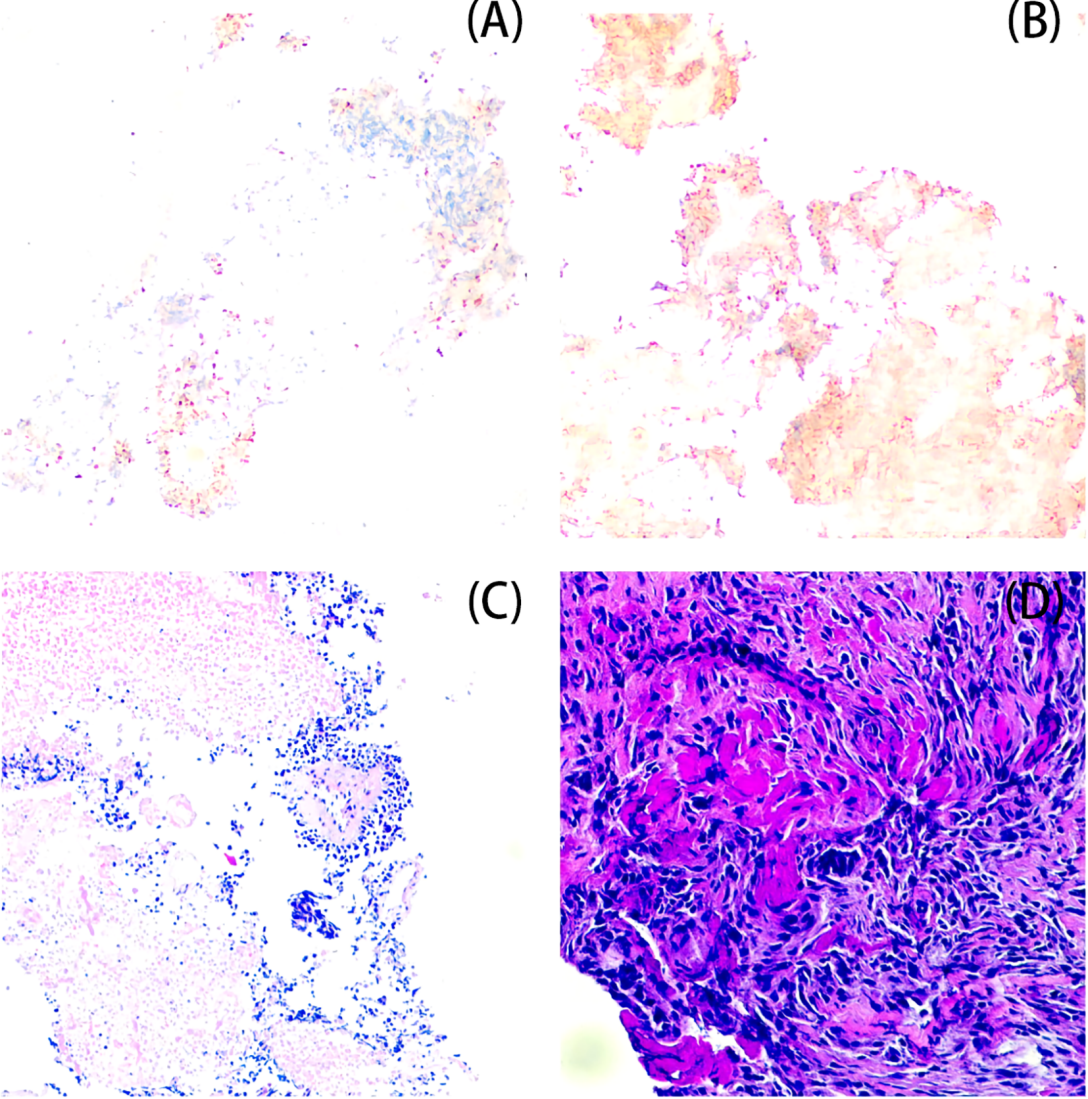
Pathology results [**(A)** INSM1(+); **(B)** Syn(+); **(C)** HE 400×; **(D)** HE 200×].

Postoperative pathology and immunohistochemical labeling confirmed the diagnosis of small-cell neuroendocrine carcinoma (SCNEC) with partial necrosis. Immunohistochemistry showed CK (a few paranuclear punctate +), Syn (+), INSM1 (+), CD99 (partially +), CD38 (-), MUM-1 (-), FLI-1 (-), CD56 (-), CgA (-), LCA (-), and Ki-67 about 80% (**Figure 3**).

Based on the pathological results, we performed a neuron-specific enolase (NSE) test, which revealed neuron-specific enolase:>370.00ng/mL. Combined with the patient’s preoperative thoracic and abdominal imaging, metastasis was ruled out. After consultation with physicians from the relevant departments, the patient was diagnosed with primary SCNEC of the gallbladder. In conclusion, systemic antitumor therapy was recommended for the patient because of the low incidence of this disease and the lack of clear treatment guidelines for it. Our hospital suggested that after recovery from surgery, the patient should be treated with the EP regimen for SCNEC (etoposide + cisplatin). Thirteen days after surgery, the patient’s digestive tract obstruction symptoms were relieved, and after full communication with the patient’s family, the patient and his family requested discharge from the hospital and transfer to an oncology hospital for further antitumor treatment. At the 1-month follow-up, the family indicated that they did not visit the relevant hospitals for follow-up treatment because of economic factors, and the patient died 3 months after discharge.

## 2 Discussion

Gallbladder cancer (GBC) constitutes 1.7% of all cancer - related fatalities (1). Neuroendocrine carcinoma of the gallbladder (GB - NEC), a rare subtype of GBC, is more malignant than GBC itself (2–4). Small - cell neuroendocrine carcinoma of the gallbladder (GB - SCNEC) is an infrequent malignant tumor with a low incidence rate. Up to now, there is no globally accepted or satisfactory treatment available. Neuroendocrine carcinoma (NEC) is a rare tumor, and the incidence of this tumor is relatively low at about 115/10 million (5). SCNECs are a poorly differentiated type of NEC. Although SCNECs are one of the most common pathological patterns of lung cancer, such tumors can appear almost anywhere in the body (6, 7). In the hepatobiliary system, primary SCNEC of the gallbladder (GB-SCNEC) and liver are extremely rare, and the limited number of publications on GB-SCNEC, consisting mainly of individual case reports or small retrospective series, adds to the complexity of diagnosis and treatment.

Gallbladder cancer (GBC) is a malignant tumor, with adenocarcinoma being the most common, accounting for 98% of all gallbladder malignancies (8). GB-SCNEC is rare in clinical practice and is often misdiagnosed. According to the National Cancer Institute (NCI), the detection methods for GB-SCNEC are similar to those for other neuroendocrine carcinomas, primarily histopathological and immunohistochemical examinations (9). Diagnosis is confirmed by microscopic observation of the morphological features of the tumor cells and detection of the expression of neuroendocrine markers, such as synaptophysin (Syn), chromogranin (CgA), and CD56. According to the US Surveillance, Epidemiology, and End Results (SEER) database, the incidence of GB-SCNEC is less than 0.74/100,000 annually, which is only 0.5% of all NECs, and the incidence of GB-SNEC is even lower. Compared with other types of neuroendocrine tumors, GB-SCNEC usually lacks a typical clinical presentation, and its symptoms, such as abdominal pain, abdominal mass, jaundice, and ascites, are mostly nonspecific. Carcinoid syndrome is extremely rare, with an incidence of less than 1% (10). Similar to neuroendocrine carcinoma, GB-SCNEC lacks specific tumor biomarkers and typical features on CT or MRI, making early diagnosis challenging (11).

Compared with other malignant tumors, SCNEC is highly aggressive, and systemic metastasis is more common, with the liver being the most common site of hematogenous metastasis, further contributing to its poor prognosis (12, 13). Currently, surgical resection is the mainstay of treatment for early-stage SCNEC and is the preferred therapeutic strategy. However, many patients are in the advanced stages of the disease at the time of diagnosis and miss the optimal time for surgery. Chemotherapy is the mainstay of treatment in these patients. According to the Guidelines for the Treatment of Neuroendocrine Carcinoma of the Digestive Tract published by the European Neuroendocrine Tumor Society (ENETS) in 2023, platinum in combination with etoposide is recommended as a first-line treatment option for patients with metastatic NEC. Irinotecan in combination with fluorouracil is a strong option for second-line treatment, and there is sufficient evidence to support this (12).

## 3 Conclusion

The literature on GB-SCNECs is limited and primarily consists of case reports and small retrospective studies. Currently, the characteristics of GB-SCNECs, including their clinicopathology and treatment, are mainly extrapolated from small-cell lung cancer. The etiology and pathogenesis of GB-SCNECs have not yet been fully elucidated. Some investigators have proposed that intestinal epithelial hyperplasia caused by chronic inflammation (including chronic cholelithiasis and cholecystitis) is a risk factor for the development of the disease because neuroendocrine cells are not present in the normal gallbladder (13–15). SCNECs exhibit highly aggressive features, including early lymph node metastasis and, most commonly, distant metastasis to the liver and lungs, leading to a poor prognosis. Currently, there is no standard treatment for GB-SCNEC. Surgery, chemotherapy, and radiotherapy are elective treatments that may improve overall survival. For patients with GB-SCNEC without serious contraindications or distant metastases, radical surgical resection is the main treatment option (16). Postoperative pathological examination and immunohistochemical staining of surgical resection specimens remain the gold standard for accurate diagnosis.

## Data Availability

The raw data supporting the conclusions of this article will be made available by the authors, without undue reservation.
